# South Western Obstetrical and Gynaecological Society

**Published:** 1991-09

**Authors:** 


### South Western Obstetrical and Gynaecological Society

### Meeting held at the Avon Gorge Hotel, Bristol, 10 May 1991

### POLYCYSTIC OVARIAN DISEASE — WIDER HEALTH ISSUES

**Robert Fox**
Bristol Maternity Hospital

The syndrome of amenorrhoea, hirsutism and obesity described by Stein and Leventhal identifies but a small proportion of women with polycystic ovarian disease (PCOD). It is now clear from ultrasound studies that 10-20% of apparently healthy women have polycystic ovaries. Defects of the insulin receptor mechanism, which lead to high circulating levels of insulin, appear to be important in the pathogenesis of the condition.

In a study of 50 women with PCOD (and 80 normal controls), we found raised median fasting insulin concentrations in both obese and non-obese cases ($p < 0.001$ and 0.01 respectively). Overall 31 (62%) of the PCOD group had fasting hyperinsulinaemia. The PCOD group had marginally higher fasting and post-carbohydrate glucose levels but none had glucose intolerance. Two subsequently developed diabetes mellitus; one during pregnancy and one during sex-steroid therapy. In contrst, 18 (36%) of the women with PCOD had a positive family history of maturity-onset diabetes compared with 7 (9%) controls ($p < 0.01$). In addition, a significantly higher proportion of both obese and non-obese patients had serum lipid abnormalities (57% cases vs 10% controls; $p < 0.001$).

These data show that a majority of women with PCOD have an abnormality of insulin state which is not primarily related to body weight. Although glucose tolerance is generally maintained, lipid abnormalities are frequently present.

### VAGINAL ULTRASOUND — THE ASSESSMENT OF EARLY FOETAL ANATOMY

**Martin Mills**
Bristol Maternity Hospital

Abdominal ultrasonography in the first trimester is largely restricted to siting of the pregnancy, assessment of viability, and measurement of the crown-rump length. By allowing closer access to the uterus and the use of higher ultrasound frequencies (>5MHz), vaginal transducers enable more detailed examination of the conceptus.

Fifty one women under forty years with defined dates of conception were scanned transvaginally at weekly intervals. A 2mm gestational sac may be visualised at 17 days post conception. It is always visible at 21 days, containing the yolk sac, measuring 4-5mm in diameter. The earliest identification of the foetal pole and heart beat was at 25 days; it was seen in all viable pregnancies by 30 days. By the fifth week, the foetal head, early foetal spine, limb buds, amnion, cord insertion and placental development are visualised. The forebrain is visible during the sixth week extending up into the empty calvarium. The mid and hind brain development occurs over the next fortnight by which time the cerebral hemispheres, falx, thalamus and cerebellum may be identified. In the eighth week, the stomach, bladder and ossification centres in the long bones, mandible and maxilla are visible. By the ninth week, the definitive spine is well visualised in both longtidunal and cross-sectional planes, and the fingers and toes may be counted. The yolk sac and physiological herniation of the mid gut (visible from the seventh week) disappears at this stage. During the tenth week, visualisation of the kidneys and a 4 chamber view of the heart is possible, although not reliably until the twelfth week.

Screening for major structural abnormalities is possible by ten weeks post conception with vaginal ultrasonography.

### ASPIRIN PREVENTS PRE-ECLAMPSIA

**Keith Louden**
Plymouth General Hospital

The rationale for the use of aspirin in hypertensive pregnancies rests upon the involvement of platelets and prostaglandins in the pathogenesis of this group of disorders. Pre-eclampsia is characterised by an imbalance in prostaglandin synthesis: production of prostacyclin, a vasodilator and inhibitor of platelet aggregation is reduced whereas production of thromboxane, with the opposing actions of vasoconstriction and promotion of platelet aggregation, is increased. The consequent platelet activation may account for some of the major clinical manifestations of pre-eclampsia. These include intra-uterine growth retardation (IUGR), the development of a coagulopathy, and the potential for damage to kidney, liver and brain.

Aspirin acts via the irreversible acetylation of cyclo-oxygenase, the enzyme which is responsible for the synthesis of thromboxane in platelets and prostacyclin in endothelial cells. The importance of low dosage is that selective inhibition of platelet thromboxane occurs, probably due to the pre-systemic inhibition of platelet cyclo-oxygenase, with little intact active aspirin reaching the systemic circulation. Hence low-dose aspirin, successful in certain aspects of cardio-vascular medicine, would appear to be a useful regime to evaluate in pre-eclampsia.

Using a longitudinal, randomised and placebo-controlled design, the effect of 60mg aspirin daily has been studied. Platelet behaviour has been measured in whole blood, using a single platelet counting technique. Serum thromboxane B2 production has been measured by radio-immunoassay. Studies were performed in non-pregnant female volunteers (NFV, $n=12$), normal primigravidae (at 28 weeks, $n=18$) and in patients with pregnancy-induced hypertension (PIH, $n=16$). Neonatal studies were performed using blood samples obtained from the umbilical cord at the time of delivery.

The table shows the results obtained, expressed as % reduction in median value after low-dose aspirin.

| | NFV | 28/52 | PIH | NORMAL NEONATE | PIH NEONATE |
|---|---|---|---|---|---|
| Arachidonic acid-induced platelet aggregation | 61 | 74 | 55 | 2* | 62 |
| Arachidonic acid-induced platelet release reaction | 100 | 93 | 97 | 12* | 17* |
| Serum thromboxane B2 production | 97 | 89 | 90 | 74 | 0* |

*indicates those changes which did not reach statistical significance, $p < 0.01$.

Profound inhibition of platelet reactivity was seen in all of the adult groups, but these findings were much less marked in the neonates exposed to maternal aspirin. It is likely that the pre-systemic acetylation of maternal platelets, with relatively little active aspirin reaching the utero-placental circulation, may account for these findings. This neonatal sparing effect may be particularly important if low-dose aspirin is to become a useful therapeutic agent for the prevention or treatment of pre-eclampsia.

Early clinical results have been very promising. A meta analysis of the available randomised placebo-controlled studies of anti-platelet therapy show that both pre-eclampsia and recurrent intra-uterine growth retardation may be prevented after treatment, and that the incidence of Caesarean section is reduced. However, there is unsufficient information available to allow any conclusions to be drawn with regard to the effect of anti-platelet therapy on perinatal mortality or the incidence of adverse neonatal effects such as intra-ventricular haemorrhage.

As early results are promising, and adverse effects appear to be minimal, there is a temptation to use low-dose aspirin in clinical practice, particularly in high risk cases. Whilst uncertainty with regard to patient selection, dose, duration of treatment, efficacy and adverse effects remains, patients should, where possible, be recruited into clinical trials such as the Collaborative Low-Dose Aspirin Studies in Pregnancy (CLASP).

### ENDOMETRIAL BUGS — WOMB AND GLOOM

**David McCoy**
Southmead Hospital

Following work in the USA claiming successful treatment of pre-menstrual tension with Doxycyline which was the subject of a "Sunday Times" article, there were a number of women requesting antibiotics for this condition. Our study was designed to show that the endometrium is microbiologically sterile.

One hundred uteri removed abdominally from pre-menopausal women were opened with a sterile scalpel and the endometrium curetted. The curettings were placed in a maintenance broth and homogenised before inoculating onto NY City Agar, Blood Agar, Fastidious Anaerobe Agar, Chocolate Agar, and an "Imagen" slide (for chlamydiae).

The results were as follows:

| Organism | No. Isolates* | Organism | No. Isolates |
|---|---|---|---|
| Lactobacillus | 11 (1) | Strep. Faecalis | 1 |
| Mycoplasma Hominis | 6 (3) | Strep. Milleri | 1 (1) |
| Staph. epidermidis | 3 (1) | E. Coli | 1 |
| Anaerobic cocci | 3 (2) | C. jeikieum | 1 |
| Kingella kingii | 2 | Ureaplasma sp. | 1 (1) |
| Moraxella urethralis | 1 | Chlamydiae | 0 |
| Strep. agalactiae | 1 | No growth | 71 |
| Strep. viridans | 1 (1) | | |

*Figures in parenthesis = no. isolates growing in the presence of one other organism.

Twenty-two percent of endometria gave positive cultures in our study. This is in contrast to some previous studies in which much higher percentages are quoted. Our method avoids contamination by cervical flora which may explain the lower isolation rate.

In conclusion the endometrium is not always sterile, a wide variety of organisms may colonize the site. Work is continuing in an attempt to define factors involved in the colonization process.

This work was done in close co-operation with Dr. Peter Cowling and Roy Marshall of Southmead Hospital.

A second short paper was presented showing work done on recording maternity statistics on a computer at St. Thomas' Hospital 25 years ago and illustrating the lack of progress that has been made over the past 25 years.

### ENDOMETRIAL RESECTION — CUT AND DRY?

**Nuala Dwyer**
Bristol Maternity Hospital

There have been few techonogical advances in gynaecology that have attracted as much interest, both in the medical profession and the media, as endometrial ablation. However, it is vital that these techniques are adequately evaluated before they are incorporated into routine clinical practice. The "gold standard" of evaluation is the randomised controlled trial. Such a trial of endometrial resection versus abdominal hysterectomy is nearing completion in the University Department of Obstetrics and Gynaecology, Bristol. 200 patients have been recruited to the trial and 185 operations have been performed. Predictably, the characteristics of the two groups is similar. 66% of the patients were aged between 35 and 44 years, they menstruated for a mean of 8 days and used 35 pads or tampons per cycle. 45% of the patients had a measured menstrual blood loss of > 80 millilitres and 40% had ferritin levels that were unrecordable. Mean operating times were 38 minutes for the endometrial resection group and 49 minutes for the hysterectomy group. These times included teaching and any other procedure performed at the same time, the most frequent being laparoscopic sterilisation. Operative complications were few but included uterine perforation and fluid overload in the resection group and bladder perforation in the hysterectomy arm. Post-operative complications were more common after hysterectomy than resection. Recovery time, including time of return to work and normal daily activities, was less in the resection group. The "failure" rate at 4 month follow-up after endometrial resection was 8% with 30% of the patients experiencing amenorrhoea and 60% hypomenorrhoea. High rates of satisfaction were found in both groups and high costs for the employer and Health Service in the hysterectomy group.

### CURRENT DILEMMAS IN ENDOMETRIOSIS

**Professor Eric Thomas**
University of Southampton

Endometriosis is being increasingly found at laparoscopy as our sensitivity to the diagnosis increases and as we realise that it has a myriad of presentations. It can now be found in peritoneum that looks completely normal at laproscopy. It has also been shown that medical treatment for endometriosis only temporarily affects the visual presentation of the disease. Finally, it has been shown that the treatment of endometriosis in infertile women does not appear to benefit their future fertility. In view of this, it is difficult to support prescription of medication for disease unless it is symptom-driven. Research should be orientated towards an understanding of the pathogenesis of the disease. Part of my research has been the establishment of an in vitro culture system for endometriosis and we have shown that endometriosis has considerable similiarities in vitro to endometrium. This supports the hypothesis of implantation, as it would be difficult to perceive such an integrated differentiation to tissue so similar to endometrium from a basic epithelium such as peritoneal mesothelium.

